# Plasma exchange and adsorption method could prevent deteriorating ARDS

**DOI:** 10.1093/omcr/omad007

**Published:** 2023-02-27

**Authors:** Yohanes WH George, Marilaeta Cindryani

**Affiliations:** Intensive Care Department, Pondok Indah Hospital, Jakarta 12310, Indonesia; Department of Anesthesiology FKUI-RSCM, RSUP Prof IGNG Ngoerah, Bali 80226, Indonesia

## INTRODUCTION

**Figure 1:**
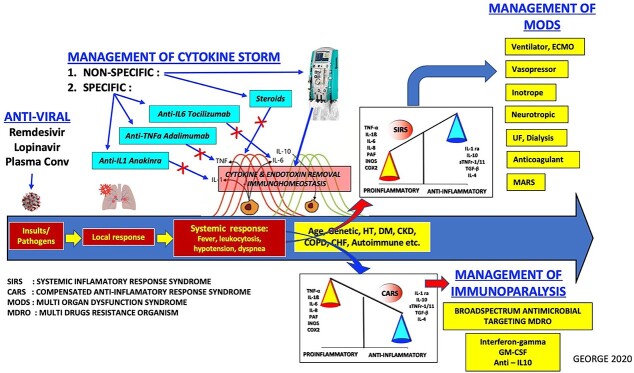
The role of extracorporeal blood purification in the management of cytokine storm in COVID ARDS.

SARS-CoV-2 infection is the best example of a disease that demonstrates the imbalance of the immune system and its poor response to vital organs. It causes myocarditis, distributive shock, Adult Respiratory Distress Syndrome (ARDS), decreased renal excretion and even prolonged post-ICU syndrome. Many therapeutic modalities have been proposed to help treat this infection to prevent multiple organ failure (Fig. 1) [1, 2].

One of advanced-scale modalities used to give the vital excretory organ time to rest is the installation of an external kidney replacement technique with the Continuous Kidney Replacement Therapy (CKRT). This organ replacement technique provides additional benefits beyond just resting the kidneys, evacuation of excess fluid, but also helps clear lactate and harmful inflammatory mediators. Moreover, coupled with the adsorption method using an innovative adsorbent coating agent on the filter, it is able to increase the clearance of unwanted interleukins, which were previously difficult to remove due to their intermediate molecular weight.

## CASE SERIES

Based on the frequencies and characteristics described in Table 1, most of our admitted patients were male and were in the range of 18–65 years.

**Table 1:** Frequencies and characteristics

Variables		Frequency (*n*)	Percent (%)
Gender	Male	20	80
	Female	5	20
Age (years)	18–65	15	60
	>65	10	40

**Figure 2:**
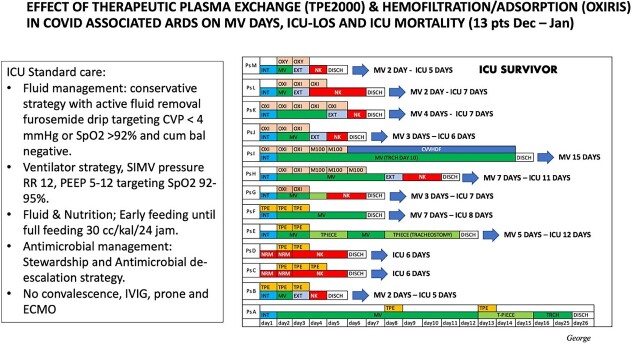
Combined therapy effect of standard care and hemofiltration technique in COVID ARDS survivors.

All of our observed 25 patients admitted to ICU in December–January 2021 were diagnosed of ARDS due to the Delta type of COVID-19. Of the 25 patients, 13 managed to survive on additional combination therapy between standard therapy with additional hemofiltration/adsorption using Oxiris™ and plasmapheresis (Fig. 2).

The standard therapy that is carried out employs conservative fluid management and active fluid removal with negative balance cumulative, ventilator strategy with PEEP 5–12 targeting SpO2 92–95%, early feeding, antimicrobial stewardship without ECMO, prone positioning or intravenous immunoglobuline.

Of the 13 surviving patients, the average length of stay was 8.8 days, whereas the length of use of the ventilator was 5.2 days (Fig. 2). This appears to be better than the 12 patients who did not survive, with the average day of death being the seventh and eighth days.

## DISCUSSION

Since its inception in the 1970s, CKRT has come a long way beyond its original intended use. The so-called Extracorporeal Blood Purification has expanded the scope of CKRT that, initially, only focused on the kidney to become an extensive effort to purify blood from unwanted waste products and inflammatory mediators. This purification role becomes prominent during the COVID-19 pandemic, where mechanical anti-inflammatory therapy is needed to improve the condition of immune system dysregulation but still maintain hemodynamic stability in critically ill patients [3, 4].

Based on Surviving Sepsis Campaign recommendation on mechanical ventilation in COVID-19 patients with ARDS, it is recommended to administer low tidal volume (4–8 mL/kgBW) with a target oxygen saturation of 92–96%, high PEEP use to improve oxygenation, traditional recruitment lung maneuvers, prone position for 12–16 hours and continuous use of muscle relaxants may be considered to achieve the desired ventilation target. An extracorporeal therapy and nitric oxide inhalation is also recommended in supporting facilities. Excess fluid is also avoided to ensure the lungs remain dry so as not to exacerbate the inflammation that interferes with ventilation and oxygenation. However, the excessive inflammatory parameter still needs to be removed by purification systems such as CKRT and plasmapheresis [5].

Unfortunately, its unavailability in many requiring hospitals and the equipment high cost makes this tool rarely accessible to those who need it. Plasmapheresis method is attempted as an intermediate option in this cost issue. Therefore, the starting point for dissemination of this external purification technique is not only based on CKRT but has incorporated plasmapheresis into it.

As seen in Fig. 2, as many as seven patients received CKRT, whereas six patients received plasmapheresis. The adsorbent (Oxiris™) was used in seven patients taking CKRT, whereas the remaining six remained on plasmapheresis, and all of them continued to receive standard therapy. The other 12 patients did not get the same opportunity for CKRT and plasmapheresis due to financial problems and availability of filters.

**Figure 3:**
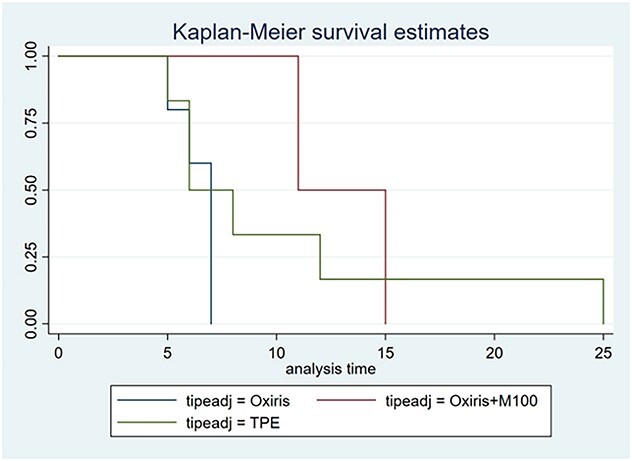
Kaplan–Meier survival analysis on the adsorbent type.

Looking into the analysis of adsorbent types in Fig. 3, we found that those survivors with Oxiris™ discharged earliest from the ICU, and the other two types such as M100 and plasmapheresis were discharged later. This simple analysis could emphasise more on the important role of early CKRT to remove cytokine in critical COVID-19 patients. Although based on the recent retrospective, a multicenter, descriptive study included 83 patients with CRS from three hospitals in Wuhan, [6] CKRT significantly reduced the inflammation; however, it did not decrease the fatality rate of patients with Cytokine Release Syndrome (CRS). Therefore, the choice of CRRT indication, dialysis time and dialysis mode should be more careful and accurate in COVID-19 patients with CRS.

Unfortunately, a small sample size, absence of specific cytokine examination and large bias of therapeutic variation, unclear baseline patient history are the weaknesses of our case series. Conclusions could not be drawn specifically from this series, but it could show the potential dual use of CKRT to manage a cytokine storm.
